# Individualized prediction of transition from subjective cognitive decline to mild cognitive impairment based on multimodal MRI: a 10-year follow-up study

**DOI:** 10.1016/j.tjpad.2025.100462

**Published:** 2026-01-01

**Authors:** Xingyan Le, Junbang Feng, Xiaoli Yu, Yuyin Wang, Qingbiao Zhang, Yuwei Xia, Feng Shi, Chuanming Li

**Affiliations:** a Medical Imaging Department, Chongqing Emergency Medical Center, Chongqing University Central Hospital, School of Medicine, Chongqing University, Chongqing, China; b Department of Research and Development, Shanghai United Imaging Intelligence, Co., Ltd. Shanghai, 200030, China

**Keywords:** Subjective cognitive decline, Mild cognitive impairment, Alzheimer’s disease, Magnetic resonance imaging, Prediction

## Abstract

**Background:**

Predicting the transition from subjective cognitive decline (SCD) to mild cognitive impairment (MCI) is critical for dementia prevention.

**Objective:**

Comprehensive assessment of MRI-based macro-/micro-structural and functional brain changes in SCD to develop an individualized model predicting transition to MCI.

**Design, Setting, and Participants:**

Patients with SCD were screened from the ADNI, NACC, and OASIS-3 databases. 89 patients met the inclusion criteria and underwent structural magnetic resonance imaging (sMRI) and resting-state functional MRI (rs-fMRI). Over a 10-year follow-up, 49 patients progressed to MCI, while 40 remained stable.

**Measurements:**

The VB-net automated brain segmentation, extracting hippocampal radiomics and whole brain subregion volume features. Brain functional features were extracted based on rs-fMRI. Cox regression was used to develop predictive models, which were independently validated with the testing set. The nomogram was constructed to estimate the probability of transition to MCI at 5-/7-/10-year. The nomogram’s accuracy was assessed using calibration curves and concordance index (C-index), and clinical utility was evaluated through decision curve analysis.

**Results:**

The model incorporating age, brain volume, functional, and radiomics features demonstrated the highest predictive performance for SCD progression in training (C-index: 0.962; 95 % CI: 0.95–0.98) and testing (C-index: 0.911; 95 % CI: 0.861–0.968) sets. A nomogram comprising 10 predictors was constructed to estimate individualized risk of progression to MCI at 5-/7-/10-year. The calibration curve showed good agreement between predicted and observed values. Decision curve analysis demonstrated the nomogram had substantial clinical value.

**Conclusions:**

This multivariate model and nomogram could accurately predict the individual progression from SCD to MCI.

## 1 Introduction

Alzheimer's disease (AD) is one of the leading causes of dementia, with a global prevalence of 4 %–7 % among individuals aged 65 and above, increasing significantly with age [1]. Early identification and timely intervention may delay AD progression and significantly improve patients’ quality of life. Subjective cognitive decline (SCD) is a preclinical stage that precedes AD and mild cognitive impairment (MCI), representing the first clinical symptom in the course of AD [2]. Patients with SCD subjectively feel a decline in memory or cognitive function, although objective neuropsychological testing shows normal results [3]. SCD carries a high risk of progression to MCI or AD, with approximately 10 %–15 % of SCD patients developing MCI annually, and 60 % of MCI patients progressing to AD within five years [4]. Investigating the progression risk and predictive strategies for SCD can support early interventions, providing a crucial "window of opportunity" for AD prevention.

Most previous research on early dementia prediction has focused on the transition from MCI to AD [5,6]. However, substantial neuronal loss and irreversible cognitive impairment have often occurred by the time MCI is diagnosed. Therefore, identifying biomarkers and prediction methods during the SCD stage holds greater clinical importance. In 2021, Yue et al. trained a machine learning model using MRI-based morphological brain features to differentiate 24 progressive SCD patients who developed MCI within seven years from 52 stable SCD patients. This study was the first to show that future cognitive decline in SCD populations could be predicted using baseline structural MRI (sMRI), although the model’s AUC was only 0.799 [7]. In 2024, Lerch et al. constructed a predictive model based on cortical thickness and subcortical volume measured by MRI. They reported a highest AUC of 0.729 for predicting progression from SCD to MCI or dementia [8].

The limited accuracy of previous models restricts their clinical applicability. A likely reason is that, as the earliest stage of AD, brain changes in SCD patients are very subtle, and macrostructural analysis may fail to capture the microscopic and functional alterations. Resting-state functional MRI (rs-fMRI) has shown value in the diagnosis and evaluation of early cognitive decline [9]. Studies have shown that rs-fMRI can detect early functional abnormalities before structural changes appear [10,11]. Kim et al. reported that combining sMRI with rs-fMRI provided additional diagnostic information compared to sMRI alone [12]. Radiomics is a novel imaging technique that captures tissue microstructure and heterogeneity. It has been widely applied in research on Parkinson’s disease and AD [13]. In a previous study, 4528 radiomics features were successfully extracted from the whole brain and combined with clinical and laboratory data to build a predictive model for progression from MCI to AD, achieving a C-index of 0.950 [14]. Until now, there is still a lack of research integrating sMRI, rs-fMRI, and radiomics to comprehensively evaluate the brain changes of SCD and predict the progression of cognitive impairment. Therefore, this study aimed to integrate sMRI and rs-fMRI, comprehensively assessing macroscopic, microscopic, and functional brain changes, and develop predictive models to forecast cognitive decline in SCD patients over 10 years.

## 2 Methods

### 2.1 Participants

Patients with SCD were screened from the Alzheimer's Disease Neuroimaging Initiative (ADNI), National Alzheimer's Coordinating Center (NACC), and Open Access Series of Imaging Studies-3 (OASIS-3) databases. The use of the above datasets was approved by the institutional review board at each site, and written consent was obtained from all participants. Among them, 89 patients met the inclusion criteria: (1) Had a SCD-related diagnostic label as baseline status; (2) Had baseline data, including age, sex, education level, body mass index (BMI), hypertension, smoking, alcohol consumption, and APOEε4 status; (3) Underwent sMRI and rs-fMRI within 1 year of baseline; (4) Availability of 10-year clinical follow-up records from baseline, with the follow-up outcome meeting either of the following conditions: (i) progression to MCI within the 10-year period, or (ii) no conversion of cognitive status throughout the entire 10-year follow-up period (i.e., remained SCD). The detailed diagnostic criteria are described in the manuals of the three databases. Briefly, SCD participants had subjective memory decline persisting for at least six months, and normal baseline objective memory performance [15,16]. MCI diagnosis required objective impairment in at least one cognitive domain, with Mini-Mental State Examination (MMSE) scores ≥24 or Montreal Cognitive Assessment (MoCA) scores ≤26. Exclusion criteria included: (1) Neurological diseases such as stroke, cerebral infarction, traumatic brain injury, and brain tumors; (2) Psychiatric or psychological disorders such as schizophrenia, intellectual disability, and depression; (3) Current significant alcohol or substance abuse. All participants were randomly assigned to training and testing sets in an 8:2 ratio. A flowchart of subject selection is shown in Supplementary Figure 1, and detailed database retrieval procedures are provided in Supplementary Table 1.

### 2.2 MRI acquisition

ADNI and NACC Datasets. Structural images were acquired from three different scanners using a 3D T1-weighted MPRAGE/IR-FSPGR sequence. Scanner 1 (Siemens 3T scanner): repetition time (TR)/echo time (TE) = 2300.0/3.0 ms, flip angle = 9°, matrix = 240 × 256, slice thickness = 1.0–1.2 mm. Scanner 2 (GE Medical Systems 3T scanner): TR/TE = 7.2–7.7/3.0–3.1 ms, flip angle = 11°, matrix = 256 × 256, slice thickness = 1.0–1.2 mm. Scanner 3 (Philips Medical Systems 3T scanner): TR/TE = 6.5–6.8/2.9–3.2 ms, flip angle = 11°, matrix = 256 × 256, slice thickness = 1.0–1.2 mm. The rs-fMRI data were acquired using standard echo-planar imaging sequences or equivalent protocols from three scanners. Scanner 1 (Siemens 3T scanner): TR/ TE= 3000.0/30.0 ms, flip angle = 80–90°, matrix = 448 × 406–448, and slice thickness = 3.3–4.4 mm. Scanner 2 (GE Medical Systems 3T scanner) used TR/TE = 3000.0/30.0 ms, flip angle = 90°, matrix = 64 × 64, and slice thickness = 3.4 mm. Scanner 3 (Philips Medical Systems 3T scanner) used TR/TE = 3000.0/30.0 ms, flip angle = 80–90°, matrix = 64 × 64, and slice thickness = 3.3–3.4 mm.

OASIS-3 Datasets. The MPRAGE structural images were acquired using two different scanners: Scanner 1 (Siemens BioGraph mMR PET-MR 3 T scanner): TR/ TE = 2.95/3.0 s, flip angle = 9°, matrix = 240 × 256, slice thickness = 1.2 mm. Scanner 2 (Siemens TIM Trio 3 T scanner): TR/ TE = 2.4/3.08–-3.16 s, flip angle = 8°, matrix = 256 × 256, slice thickness = 1.0 mm. The rs-fMRI data were acquired using standard echo-planar imaging sequences from two scanners. Scanner 1 (Siemens BioGraph mMR PET-MR 3 T scanner): TR/ TE = 2200/27 ms, flip angle = 90°, matrix = 64 × 64, slice thickness = 4.0 mm. Scanner 1 (Siemens TIM Trio 3 T scanner): TR/ TE = 2200–2500/27 ms, flip angle = 90°, matrix = 64 × 64, slice thickness = 4.0 mm.

### 2.3 Brain segmentation and subregions volume measurement

A 3D VB-net deep learning model was used to segment the brain based on the Automated Anatomical Labeling (AAL) atlas and to measure the volumes of subregions. MRI data were preprocessed by applying rotation, resampling, resizing, skull stripping, bias field correction, histogram matching, and gray-scale normalization [[17], [18], [19], [20]]. The segmentation model utilized an end-to-end deep convolutional neural network trained with paired MRI images and corresponding anatomical labels. The model was trained using data from 1800 participants, achieving an average Dice similarity coefficient of 0.92 [21]. The network segmented the brain into 90 cortical and subcortical regions and 26 cerebellar regions and automatically measured their volumes (Supplementary Table 2).

### 2.4 Rs-fMRI data analysis and functional features extraction

Firstly, all MR images were converted from DICOM format to Neuroimaging Informatics Technology Initiative (NIFTI). Preprocessing was conducted in MATLAB 2022b and included the following steps: (1) Removal of the first 10 time points to allow for signal stabilization; (2) Slice timing correction to align acquisition times across slices; (3) Head motion correction; (4) Spatial normalization to Montreal Neurological Institute (MNI) space and resampling to 3 × 3 × 3 mm³ voxels; (5) Spatial smoothing using a 6 mm full width at half maximum (FWHM) Gaussian kernel; (6) Linear trend removal; (7) Regressing out the influence of head movement (Friston 24 parameter model), the cerebrospinal fluidsignal noise, and the white matter signal noise from the fMRI; (8) Band-pass filtering (0.01–0.08 Hz). The entire brain was parcellated into 116 regions of interest (ROIs) based on the AAL atlas, and the functional features were calculated using the RESTplus toolkit (v1.25) [22] . The square root of the power spectrum was averaged across the 0.01–0.08 Hz band to compute the amplitude of low-frequency fluctuations (ALFF) [23] . The ratio of the sum of amplitude within the low-frequency band (i.e., ALFF) to that of the entire frequency band was calculated as fALFF [24] . For regional homogeneity (ReHo), the Kendall’s coefficient of concordance (KCC) was calculated for each voxel and its 26 neighboring voxels [25] . Using the functional connectivity analysis module, the Pearson correlation coefficient was used to calculate the functional connectivity (FC) between each ROI and all other voxels in the brain. To standardize the data for subsequent analysis, Fisher's z-transformation was applied to the FC values to produce zFC maps [26] . The mean zFC value between each ROI and all other voxels was then extracted [27,28].

### 2.5 Radiomics features of bilateral hippocampus extraction

A total of 4528 radiomics features were extracted from the left and right hippocampus of each patient. These features were grouped into four categories: intensity, shape, texture (including Gray Level Co-occurrence Matrix [GLCM], Gray Level Run Length Matrix [GLRLM], Gray Level Size Zone Matrix [GLSZM], Neighboring Gray Tone Difference Matrix [NGTDM], and Gray Level Dependence Matrix [GLDM]), and high-level features. High-level features were derived from 24 filters including Box Mean, additive Gaussian noise, binomial blur, curvature flow, Box-Sigma, normalization, Laplace sharpening, discrete Gaussian, mean, speckle noise, recursive Gaussian, shot noise, and Laplacian of Gaussian (LoG) with sigma values of 0.5, 1, 1.5, and 2. Wavelet transformations were also applied. All radiomics features were normalized using z-score transformation.

### 2.6 Feature selection and model construction

Univariable Cox regression analysis was used to separately determine clinical and brain volume indicators associated with SCD-to-MCI conversion. Radiomics and functional features were further selected using the Least Absolute Shrinkage and Selection Operator (LASSO) method, which was suitable for high-dimensional data. The radiomics score and functional score for each patient were calculated by a linear combination of the chosen features multiplied by their respective coefficients.

**Fig. 1:**
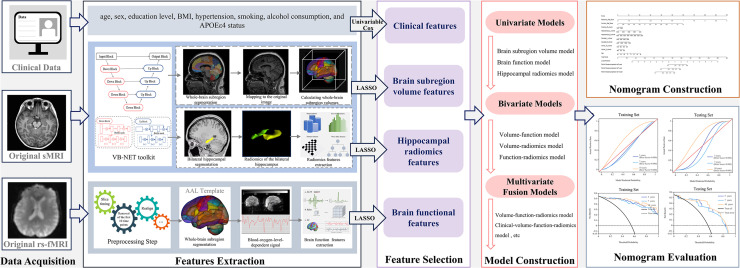
The workflow diagram of this study.

Cox regression models were constructed based on volume, radiomics, functional, and combined features. Each model was independently validated using the testing set. Model performance was evaluated using the concordance index (C-index) with a 95 % confidence interval (CI). A multipredictor nomogram was constructed to provide individualized risk estimates. Kaplan-Meier survival analyses were used to examine the association between model outputs and time to progression from SCD to MCI. Calibration curves and decision curve analysis (DCA) were used to assess the clinical utility of the nomogram. The analytical workflow was presented in Fig. 1.

### 2.7 Statistical analysis

All statistical analyses were performed using R software (v3.6.0) and Python (v3.10.6). For continuous variables, the Student’s *t*-test was applied to normally distributed data, and the Mann-Whitney U test was used for non-normal distributions. The chi-square test was used for categorical variables. A p-value < 0.05 was considered statistically significant. Feature standardization, selection, and model construction were conducted using the uAI Research Portal (Version: 20,220,230).

## 3 Results

A total of 89 patients with SCD were included in this study, comprising 17 from the ADNI database, 3 from the NACC database, and 69 from the OASIS-3 database. No significant differences in clinical characteristics were observed between the training and testing groups (Supplementary Table 3). During the 10-year follow-up, 49 patients progressed to MCI, while 40 remained cognitively stable. The cumulative progression rates at 5, 7, and 10 years were 39.32 % (35/89), 47.19 % (42/89), and 55.06 % (49/89), respectively.

**Fig. 2:**
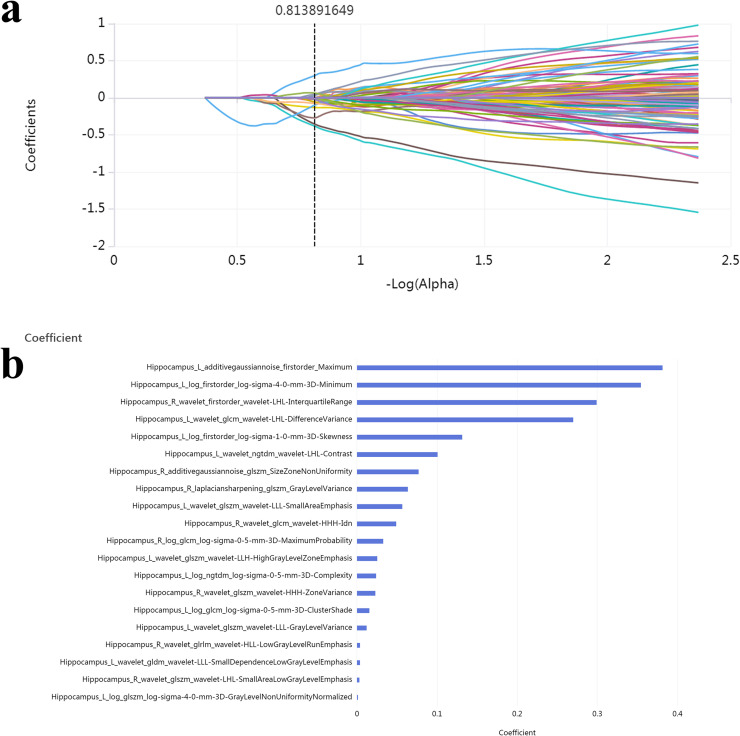
Radiomics feature Selection Using LASSO Regression in the Training Set. (a) The Lasso path displayed coefficient profiles for radiomic features across the entire range of possible values; (b) 20 optimal radiomics features were selected using the LASSO method.

Univariable Cox regression analysis identified age as an independent clinical risk factor for progression from SCD to MCI. Among the brain volume features, the volumes of the right putamen, bilateral hippocampus, bilateral caudate, and cerebellar vermis IX–X were significantly associated with disease progression (*P* < 0.05) (Table 1). For radiomics features, 20 features were retained after dimensionality reduction using LASSO regression, including 3 intensity features, 15 texture features, and 2 wavelet-based texture features (Fig. 2). Similarly, 8 functional features were selected, including ALFF in the right cerebellar lobule VI, right cerebellar lobule III, vermis lobule III, orbital part of the left middle frontal gyrus, left thalamus, and right lingual gyrus; fALFF in the left temporal pole-middle temporal gyrus; and ReHo in the left parahippocampal gyrus. Radiomics scores and functional scores for each patient were then calculated (Supplementary Table 4).

**Table 1:** Univariable Cox regression analysis of clinical characteristics and brain volume features in the training set. * indicates a significant P value (*P* < 0.05).

	**Variables**	**HR**	**95 % CI**	**P value**
Clinical characteristics	Age	1.0905	1.0332 ∼ 1.1510	0.0018*
	Gender	1.8023	0.9583 ∼ 3.3895	0.0690
	Education	1.0214	0.9008 ∼ 1.1582	0.7423
	BMI	1.0165	0.9537 ∼ 1.0834	0.6170
	Hypertension	1.5668	0.8223 ∼ 2.9854	0.1744
	Smoke	0.7007	0.3610 ∼ 1.3602	0.2957
	Drink	0.7079	0.1716 ∼ 2.9196	0.6345
	APOEε4	1.2184	0.6084 ∼ 2.4401	0.5791
				
Brain volume features retained	Putamen_R_volume	1.0005	1.00004∼1.00101	0.0351*
	Hippocampus_L_volume	1.0004	1.00005∼1.00072	0.0249*
	Hippocampus_R_volume	1.0005	1.00008∼1.00091	0.0197*
	Caudate_L_volume	1.0007	1.00011∼1.00124	0.0183*
	Vermis_9_volume	1.0040	1.00112∼1.00681	0.0063*
	Caudate_R_volume	1.0010	1.00031∼1.00171	0.0046*
	Vermis_10_volume	1.0048	1.00149∼1.0082	0.0046*

HR = hazard ratio; CI = confidence interval; BMI= body mass index; *R*=right; *L*=left.

In the training cohort, the C-index values of the models established with features of volume, function, radiomics, volume-function, volume-radiomics, function-radiomics, volume-function-radiomics, clinical-volume-function-radiomics were as follows: 0.702(95 % CI:0.597–0.842), 0.816(95 % CI:0.784–0.854), 0.921(95 % CI:0.903–0.941), 0.862(95 % CI:0.837–0.892), 0.933(95 % CI:0.916–0.952), 0.941(95 % CI:0.923–0.959), 0.947(95 % CI:0.933–0.965), 0.962(95 % CI:0.95–0.98). In the testing set, the C-index values of the models established with features of volume, function, radiomics, volume-function, volume-radiomics, function-radiomics, volume-function-radiomics, clinical-volume-function-radiomics were as follows: 0.664(95 % CI:0.616–0.719), 0.71(95 % CI:0.632–0.804), 0.863(95 % CI:0.806–0.929), 0.782(95 % CI:0.676–0.895), 0.863(95 % CI:0.791–0.935), 0.871(95 % CI:0.809–0.935), 0.903(95 % CI:0.861–0.942), 0.911(95 % CI:0.861–0.968) (Table 2). Among these models, the clinical-radiomics-volume-function model had the best performance (*P* < 0.05). Kaplan-Meier survival curves showed that the survival outcomes in the high-risk group were significantly worse than those in the low-risk group (Supplementary Figure 2).

**Table 2:** Prediction performance of different models in training set and testing set.

	**Training Set**				**Testing Set**		
**Models**	C-index	Lower	Upper		C-index	Lower	Upper
Brain subregion volume	0.702	0.597	0.842		0.664	0.616	0.719
Brain function	0.816	0.784	0.854		0.71	0.632	0.804
Hippocampal radiomics	0.921	0.903	0.941		0.863	0.806	0.929
Volume-function	0.862	0.837	0.892		0.782	0.676	0.895
Volume-radiomics	0.933	0.916	0.952		0.863	0.791	0.935
Function- radiomics	0.941	0.923	0.959		0.871	0.809	0.935
Volume-function-radiomics	0.947	0.933	0.965		0.903	0.861	0.942
Clinical-volume-function	0.862	0.835	0.894		0.782	0.676	0.893
Clinical-volume-radiomics	0.935	0.918	0.954		0.887	0.836	0.946
Clinical-function-radiomics	0.941	0.924	0.959		0.871	0.809	0.935
Clinical-volume-function-radiomics	0.962	0.95	0.98		0.911	0.861	0.968

**Fig. 3:**
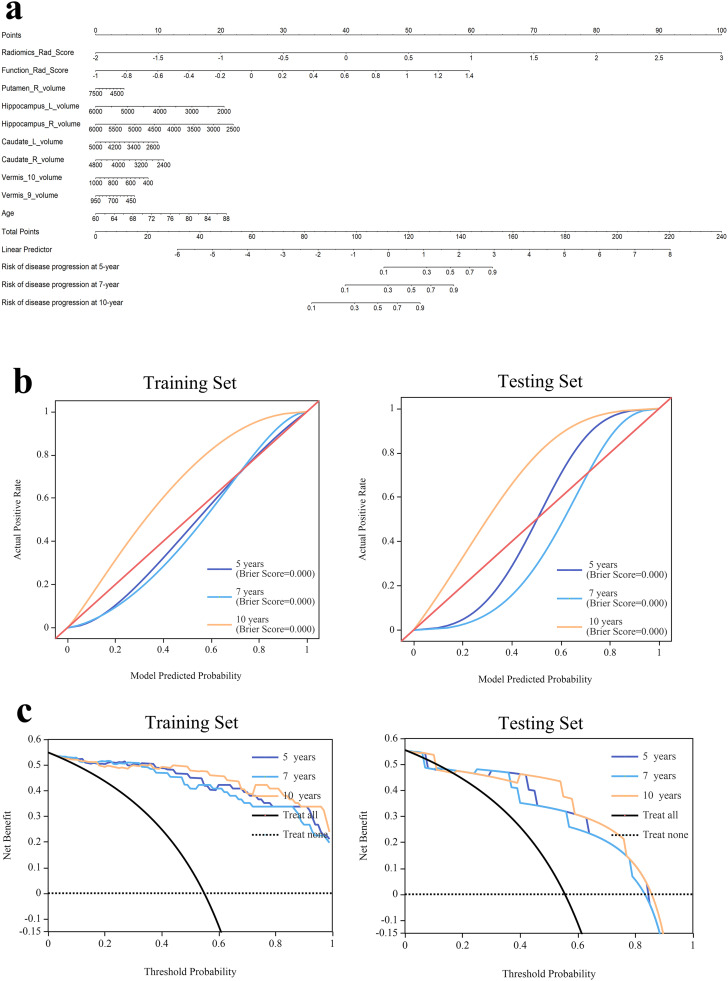
The nomogram, calibration curves and DCA of the model based on the clinical-radiomics-volume-function model. (a) The nomogram of the clinical-volume-function-radiomics model. (b) The calibration curves for nomogram goodness of fit in the training set and testing set. The 45°line indicated that the probability predicted by the model matches the actual probability. The closer the distance between the 2 curves was, the higher the accuracy. (c) The DCA of different models in the training set and testing set.

Based on the clinical-volume-function-radiomics model, a nomogram was developed incorporating 10 independent prognostic factors: Radiomics_Rad_Score, Function_Rad_Score, volumes of the right putamen, left and right hippocampus, left and right caudate, vermis lobules IX and X, and age. The nomogram was used to estimate progression probabilities at 5, 7, and 10 years (Fig. 3a). Calibration curves demonstrated excellent agreement between predicted and observed outcomes (Fig. 3b), and the decision curve analysis showed a higher net benefit within a reasonable range of threshold probabilities in both training and testing sets (Fig. 3c).

## 4 Discussion

AD poses a growing public health challenge in aging populations. SCD represents the preclinical stage of AD, and predicting its progression risk is critical for early intervention and improving prognosis. However, effective prediction tools and methods are currently lacking in clinical practice. Rs-fMRI reflects early functional abnormalities in neural activity, sMRI reveals morphological changes in different brain regions, and radiomics captures microscopic structural alterations invisible to the human eye. To the best of our knowledge, this study was the first to integrate rs-fMRI, sMRI, and radiomics features using artificial intelligence to build predictive models for cognitive decline in SCD.

In this study, we found that combining age, brain volume, radiomics, and functional features yielded the best predictive performance. The C-index of this model was 0.962 in the training set and 0.911 in the testing set. The calibration and decision curves demonstrated good stability and clinical utility, indicating that this model could accurately predict early cognitive decline in SCD. Although several studies have used artificial intelligence and MRI to predict progression from MCI to dementia, relatively few have targeted the SCD-to-MCI transition [7,[8], [9], [10], [11], [12], [13], [14]]. Compared to previous reports, our model achieved the highest accuracy. One reason may be that earlier studies primarily focused on sMRI features such as brain volume and cortical thickness, overlooking more sensitive indicators like radiomics features and functional network alterations. Our model is entirely MRI-based, safe, and non-invasive, avoiding the need for cerebrospinal fluid or blood sampling and the radiation associated with PET imaging, making it highly suitable for routine clinical use.

A VB-net model was used to automatically segment the brain into 90 cortical and subcortical regions and 26 cerebellar regions based on 3D T1-weighted images, and to calculate the volume of each region. This model was developed based on the classic V-net architecture for 3D object segmentation, which was an encoder-decoder network with skip and residual connections. VB-Net was further improved in reducing the computational complexity of the model by the addition of bottleneck layers. The optimized architecture of VB-Net achieves a favorable balance between accuracy, efficiency, and clinical applicability [17]. This network has been successfully applied to the study of various brain disorders, such as Parkinson's disease and MCI, showing strong performance [[21], [22], [23], [24], [25], [26], [27], [28], [29]]. In our analysis, volumes of the right putamen, bilateral caudate, cerebellar vermis IX–X, and bilateral hippocampus were significantly associated with progression from SCD to MCI, suggesting that atrophy in these regions may play a key role in cognitive decline. The putamen and caudate are components of the basal ganglia and are involved in motor control, cognition, and emotion [30] . Changes in their volumes have been linked to early cognitive impairment in SCD [31,32] . The cerebellar vermis is involved in spatial orientation through the cerebellar cortical loop [33], and reduced gray matter volume in this region has been found associated with cognitive impairment in SCD [34] . The hippocampus, located between the thalamus and the medial temporal lobe, plays a critical role in memory storage. Hippocampal atrophy is a well-established early marker of AD [35], and its changes in SCD may be related to cholinergic system dysfunction [36] .

This study was the first to use hippocampal radiomics and functional parameters to predict cognitive progression in SCD. Among the 20 selected radiomics features, there were 3 intensity features, 15 texture features, and 2 wavelet-based texture features. Intensity features reflect the grayscale of the image and can indicate local neuron density [37] . Texture features represent image heterogeneity and complexity, which may result from microscopic brain changes such as amyloid-beta plaques and neurofibrillary tangles [38] .These features provide more detailed information than volume. In addition to structural changes, alterations in ALFF, fALFF, and ReHo values in specific brain regions also showed strong predictive value. ALFF and fALFF reflect the level of spontaneous brain activity from an energy perspective by detecting the amplitude of relative baseline fluctuations in brain functional activity signals [39] . ReHo reflects the synchronicity of spontaneous activity between local and peripheral voxels in the brain by analyzing a series of rs-fMRI signals from voxels [25] . Previous studied had shown that SCD patients exhibit higher ALFF values in the bilateral lingual and frontal gyri compared to MCI patients [40,41] . Han et al. reported increased ALFF activity in the right posterior cerebellum in SCD patients compared to controls [42], and enhanced ALFF in the thalamus had also been observed in SCD [43] .

In this study, we also examined the relationship between clinical factors and cognitive decline in patients with SCD. The results showed that only age had predictive value and was included in the final model. Previous studies had demonstrated that age was closely associated with the progression of SCD [44] . The older SCD patients were, the higher their risk of developing MCI. Patients older than 65 years tended to exhibit more severe amyloid deposition and neuronal damage than those younger than 65 years [45] . Although previous studies had identified sex, education level, and the APOEε4 allele as important factors contributing to cognitive decline [46,47], our study did not find predictive significance for education level or APOEε4 allele. A possible explanation was that these factors may play different roles at various stages of cognitive impairment [3]. During the transition from SCD to MCI, these factors may not be as critical as changes in brain function and structure.

This study had several limitations. First, although the follow-up period extended to ten years, the sample size was relatively small. Second, despite integrating data from three databases and using internal validation to enhance model robustness, external validation was not conducted due to data source limitations. Third, this study only analyzed baseline data from SCD participants and did not assess longitudinal changes in relevant features. Future studies should include larger sample sizes and conduct external independent validation.

Altogether, this was the first comprehensive MRI-based study to evaluate macroscopic, microscopic, and functional brain changes in SCD patients with a 10-year follow-up, which is the longest reported to date. We developed a predictive model that accurately estimated the risk of progression from SCD to MCI at 5, 7, and 10 years. High-risk individuals with SCD could be identified and treated early to delay the onset of AD and improve clinical outcomes. Our findings offer a feasible and objective early prediction tool with significant clinical utility.

## Ethical standards

Ethics approval was obtained by the ADNI, NACC, and OASIS-3 investigators from the local ethical committees of all involved sites. Access to ADNI (https://adni.loni.usc.edu/data-samples/access-data/), NACC (https://naccdata.org/), and OASIS-3 (https://www.nitrc.org/projects/oasis3/) data was granted to the investigators of the current study after registration to each respective database and compliance with their data usage agreements. The study was conducted in accordance with the Declaration of Helsinki and all study participants provided written informed consent for data collection. All work complied with ethical regulations for work with human participants.

## Declaration of generative AI and AI-assisted technologies in the writing process

We did not use generative AI to generate any manuscript text, but help polish some sentences.

## Declaration of competing interest

The authors declare no conflicts of interest.

## Author contributions

Chuanming Li, Xingyan Le and Junbang Feng: conceptualization. Xingyan Le, Junbang Feng, Xiaoli Yu: data curation. Yuyin Wang and Qingbiao Zhang: data preprocessing. Xingyan Le and Qingbiao Zhang: computer programming. Yuwei Xia and Feng Shi: technical consultants. Xingyan Le and Junbang Feng: data analysis. Chuanming Li, Xingyan Le and Junbang Feng: manuscript drafting. Chuanming Li and Junbang Feng: manuscript editing. All authors approved the submitted version.

## Funding

This work was supported by the 10.13039/501100012226Fundamental Research Funds for the Central Universities (Project NO. 2022CDJYGRH-004); the 10.13039/501100004374Chongqing Medical Scientific Research Project (Joint project of Chongqing Health Commission and Science and Technology Bureau) (2026MSXM001); the 10.13039/501100013148Science and Technology Research Program of Chongqing Municipal Education Commission (Grant No. KJQN202400117).

## CRediT authorship contribution statement

**Xingyan Le:** Writing – review & editing, Writing – original draft, Validation, Resources, Methodology, Investigation, Formal analysis, Data curation. **Junbang Feng:** Writing – review & editing, Writing – original draft, Resources, Funding acquisition, Formal analysis. **Xiaoli Yu:** Validation, Investigation, Data curation. **Yuyin Wang:** Resources, Methodology. **Qingbiao Zhang:** Visualization, Software. **Yuwei Xia:** Resources. **Feng Shi:** Resources. **Chuanming Li:** Writing – review & editing, Writing – original draft, Validation, Methodology, Investigation.

## Declaration of competing interest

The authors declare that they have no known competing financial interests or personal relationships that could have appeared to influence the work reported in this paper.

## Appendix Supplementary materials

Image, application 1
