# Supplementary material for: Individualized prediction of transition from subjective cognitive decline to mild cognitive impairment based on multimodal MRI: a 10-year follow-up study
Source: J Prev Alzheimers Dis. 2026 Jan 1;13(2):100462. doi: 10.1016/j.tjpad.2025.100462 (PMC12869052; doi:10.1016/j.tjpad.2025.100462)

**Supplementary Online Content**

**Supplementary Table 1：**The search process of the Alzheimer's Disease Neuroimaging Initiative (ADNI), Open Access Series of Imaging Studies-3 (OASIS-3), and National Alzheimer's Coordinating Center (NACC) databases.

**Supplementary Table 2：**The full name of 116 brain regions of automated anatomical labeling template (AAL).

**Supplementary Table 3：**Baseline characteristics of the SCD patients in the training set and testing set.

**Supplementary Table 4：**Final feature sets across all models.

**Supplementary Figure 1：**The flowchart of patient selection.

**Supplementary Figure 2：**Kaplan-Meier curve of transition time based on the clinical-volume-function-radiomics model: training set (a) and testing set (b).

**Supplementary Table 1：**The search process of the Alzheimer's Disease Neuroimaging Initiative (ADNI), Open Access Series of Imaging Studies-3 (OASIS-3), and National Alzheimer's Coordinating Center (NACC) databases.

| **Name of database** | **Research procedure** |
| --- | --- |
| ADNI | (1) SCD: Participants are required to have an SMC diagnostic label at baseline. All participants had ten-year follow-up records, with conversion to MCI as the study endpoint.  (2) MCI: Participants were required to have an MCI diagnostic label at baseline, including MCI, LMCI, and EMCI. Additionally, participants must have MMSE scores ≥24 or MoCA scores ≤26.  We subsequently excluded recordings without any matched T1WI and rs-fMRI images within one year of their clinical diagnosis. If multiple images were available, only the one closest in time to the diagnosis was chosen. To obtain more detailed diagnostic criteria, please refer to the procedure manual of ADNI (http://www.adni-info.org). |
| OASIS-3 | (1) SCD: we initially selected normal cognition based on the clinical assessments (i.e., NORMCOG=1). Based on their DECSUB values, the recordings were categorized into SCD (i.e., DECSUB =1) groups, with those missing such values being excluded. In cases where multiple recordings from the same individual were present in the SCD groups, only those in the latter group were retained.  (2) MCI: We initially selected MCI based on the clinical assessments (i.e., MCIAPLUS=1, MCIAMEM=1, MCINON1=1, MCINON2=1). In cases where multiple recordings from the same individual were present in the MCI groups, only those in the latter group were retained. Additionally, participants must have MMSE scores ≥24 or MoCA scores ≤26.  We subsequently excluded recordings without any matched T1WI and rs-fMRI images within one year of their clinical diagnosis. If multiple images were available, only the one closest in time to the diagnosis was chosen. To obtain more detailed diagnostic criteria, please refer to the procedure manual of OASIS-3 (https://www.nitrc.org/projects/oasis3/). |
| NACC | (1) SCD: we initially selected normal cognition based on the clinical assessments (i.e., NACCUDSD=1). Based on their DECSUB values, the recordings were categorized into SCD (i.e., DECSUB =1) groups, with those missing such values being excluded. In cases where multiple recordings from the same individual were present in the SCD groups, only those in the latter group were retained.  (2) MCI: We initially selected MCI based on the clinical assessments (i.e., NACCUDSD=3). In cases where multiple recordings from the same individual were present in the MCI groups, only those in the latter group were retained. Additionally, participants must have MMSE scores ≥24 or MoCA scores ≤26.  We subsequently excluded recordings without any matched T1WI and rs-fMRI images within one year of their clinical diagnosis. If multiple images were available, only the one closest in time to the diagnosis was chosen. To obtain more detailed diagnostic criteria, please refer to the procedure manual of NACC (https://naccdata.org/). |

**Supplementary Table 2：**The full name of 116 brain regions of automated anatomical labeling template (AAL).

| **Labels** | **Regions** | **Abbreviation** | **Side** |
| --- | --- | --- | --- |
| 1 | Precental gyrus | Precentral_L | Left |
| 2 | Precental gyrus | Precentral_R | Right |
| 3 | Superior frontal gyrus, dorsolateral | Frontal_Sup_L | Left |
| 4 | Superior frontal gyrus, dorsolateral | Frontal_Sup_R | Right |
| 5 | Superior frontal gyrus, orbital part | Frontal_Sup_Orb_L | Left |
| 6 | Superior frontal gyrus, orbital part | Frontal_Sup_Orb_R | Right |
| 7 | Middle frontal gyrus | Frontal_Mid_L | Left |
| 8 | Middle frontal gyrus | Frontal_Mid_R | Right |
| 9 | Middle frontal gyrus, orbital part | Frontal_Mid_Orb_L | Left |
| 10 | Middle frontal gyrus, orbital part | Frontal_Mid_Orb_R | Right |
| 11 | Inferior frontal gyrus, opercular part | Frontal_Inf_Oper_L | Left |
| 12 | Inferior frontal gyrus, opercular part | Frontal_Inf_Oper_R | Right |
| 13 | Inferior frontal gyrus, triangular part | Frontal_Inf_Tri_L | Left |
| 14 | Inferior frontal gyrus, triangular part | Frontal_Inf_Tri_R | Right |
| 15 | Inferior frontal gyrus, orbital part | Frontal_Inf_Orb_L | Left |
| 16 | Inferior frontal gyrus, orbital part | Frontal_Inf_Orb_R | Right |
| 17 | Rolandic operculum | Rolandic_Oper_L | Left |
| 18 | Rolandic operculum | Rolandic_Oper_R | Right |
| 19 | Supplementary motor area | Supp_Motor_Area_L | Left |
| 20 | Supplementary motor area | Supp_Motor_Area_R | Right |
| 21 | Olfactory cortex | Olfactory_L | Left |
| 22 | Olfactory cortex | Olfactory_R | Right |
| 23 | Superior frontal gyrus, medial | Frontal_Sup_Medial_L | Left |
| 24 | Superior frontal gyrus, medial | Frontal_Sup_Medial_R | Right |
| 25 | Superior frontal gyrus, medial orbital | Frontal_Med_Orb_L | Left |
| 26 | Superior frontal gyrus, medial orbital | Frontal_Med_Orb_R | Right |
| 27 | Gyrus rectus | Rectus_L | Left |
| 28 | Gyrus rectus | Rectus_R | Right |
| 29 | Insula | Insula_L | Left |
| 30 | Insula | Insula_R | Right |
| 31 | Anterior cingulate and paracingulate gyri | Cingulum_Ant_L | Left |
| 32 | Anterior cingulate and paracingulate gyri | Cingulum_Ant_R | Right |
| 33 | Median cingulate and paracingulate gyri | Cingulum_Mid_L | Left |
| 34 | Median cingulate and paracingulate gyri | Cingulum_Mid_R | Right |
| 35 | Posterior cingulate gyrus | Cingulum_Post_L | Left |
| 36 | Posterior cingulate gyrus | Cingulum_Post_R | Right |
| 37 | Hippocampus | Hippocampus_L | Left |
| 38 | Hippocampus | Hippocampus_R | Right |
| 39 | Parahippocampal gyrus | ParaHippocampal_L | Left |
| 40 | Parahippocampal gyrus | ParaHippocampal_R | Right |
| 41 | Amygdala | Amygdala_L | Left |
| 42 | Amygdala | Amygdala_R | Right |
| 43 | Calcarine fissure and surrounding cortex | Calcarine_L | Left |
| 44 | Calcarine fissure and surrounding cortex | Calcarine_R | Right |
| 45 | Cuneus | Cuneus_L | Left |
| 46 | Cuneus | Cuneus_R | Right |
| 47 | Lingual gyrus | Lingual_L | Left |
| 48 | Lingual gyrus | Lingual_R | Right |
| 49 | Superior occipital gyrus | Occipital_Sup_L | Left |
| 50 | Superior occipital gyrus | Occipital_Sup_R | Right |
| 51 | Middle occipital gyrus | Occipital_Mid_L | Left |
| 52 | Middle occipital gyrus | Occipital_Mid_R | Right |
| 53 | Inferior occipital gyrus | Occipital_Inf_L | Left |
| 54 | Inferior occipital gyrus | Occipital_Inf_R | Right |
| 55 | Fusiform gyrus | Fusiform_L | Left |
| 56 | Fusiform gyrus | Fusiform_R | Right |
| 57 | Postcentral gyrus | Postcentral_L | Left |
| 58 | Postcentral gyrus | Postcentral_R | Right |
| 59 | Superior parietal gyrus | Parietal_Sup_L | Left |
| 60 | Superior parietal gyrus | Parietal_Sup_R | Right |
| 61 | Inferior parietal, but supramarginal and angular gyri | Parietal_Inf_L | Left |
| 62 | Inferior parietal, but supramarginal and angular gyri | Parietal_Inf_R | Right |
| 63 | Supramarginal gyrus | SupraMarginal_L | Left |
| 64 | Supramarginal gyrus | SupraMarginal_R | Right |
| 65 | Angular gyrus | Angular_L | Left |
| 66 | Angular gyrus | Angular_R | Right |
| 67 | Precuneus | Precuneus_L | Left |
| 68 | Precuneus | Precuneus_R | Right |
| 69 | Paracentral lobule | Paracentral_Lobule_L | Left |
| 70 | Paracentral lobule | Paracentral_Lobule_R | Right |
| 71 | Caudate nucleus | Caudate_L | Left |
| 72 | Caudate nucleus | Caudate_R | Right |
| 73 | Lenticular nucleus, putamen | Putamen_L | Left |
| 74 | Lenticular nucleus, putamen | Putamen_R | Right |
| 75 | Lenticular nucleus, pallidum | Pallidum_L | Left |
| 76 | Lenticular nucleus, pallidum | Pallidum_R | Right |
| 77 | Thalamus | Thalamus_L | Left |
| 78 | Thalamus | Thalamus_R | Right |
| 79 | Heschl gyrus | Heschl_L | Left |
| 80 | Heschl gyrus | Heschl_R | Right |
| 81 | Superior temporal gyrus | Temporal_Sup_L | Left |
| 82 | Superior temporal gyrus | Temporal_Sup_R | Right |
| 83 | Temporal pole: superior temporal gyrus | Temporal_Pole_Sup_L | Left |
| 84 | Temporal pole: superior temporal gyrus | Temporal_Pole_Sup_R | Right |
| 85 | Middle temporal gyrus | Temporal_Mid_L | Left |
| 86 | Middle temporal gyrus | Temporal_Mid_R | Right |
| 87 | Temporal pole: middle temporal gyrus | Temporal_Pole_Mid_L | Left |
| 88 | Temporal pole: middle temporal gyrus | Temporal_Pole_Mid_R | Right |
| 89 | Inferior temporal gyrus | Temporal_Inf_L | Left |
| 90 | Inferior temporal gyrus | Temporal_Inf_R | Right |
| 91 | cerebellum | Cerebelum_Crus1_L | Left |
| 92 | cerebellum | Cerebelum_Crus1_R | Right |
| 93 | cerebellum | Cerebelum_Crus2_L | Left |
| 94 | cerebellum | Cerebelum_Crus2_R | Right |
| 95 | cerebellum | Cerebelum_3_L | Left |
| 96 | cerebellum | Cerebelum_3_R | Right |
| 97 | cerebellum | Cerebelum_4_5_L | Left |
| 98 | cerebellum | Cerebelum_4_5_R | Right |
| 99 | cerebellum | Cerebelum_6_L | Left |
| 100 | cerebellum | Cerebelum_6_R | Right |
| 101 | cerebellum | Cerebelum_7b_L | Left |
| 102 | cerebellum | Cerebelum_7b_R | Right |
| 103 | cerebellum | Cerebelum_8_L | Left |
| 104 | cerebellum | Cerebelum_8_R | Right |
| 105 | cerebellum | Cerebelum_9_L | Left |
| 106 | cerebellum | Cerebelum_9_R | Right |
| 107 | cerebellum | Cerebelum_10_L | Left |
| 108 | cerebellum | Cerebelum_10_R | Right |
| 109 | cerebellum | Vermis_1_2 | / |
| 110 | cerebellum | Vermis_3 | / |
| 111 | cerebellum | Vermis_4_5 | / |
| 112 | cerebellum | Vermis_6 | / |
| 113 | cerebellum | Vermis_7 | / |
| 114 | cerebellum | Vermis_8 | / |
| 115 | cerebellum | Vermis_9 | / |
| 116 | cerebellum | Vermis_10 | / |

**Supplementary Table 3：**Baseline characteristics of the SCD patients in the training set and testing set.

| **Variable** | |  | | **Training Set(n=71)** | **Testing Set(n=18)** | **p-value** |
| --- | --- | --- | --- | --- | --- | --- |
| Age (Median[Q1,Q3]) |  | | | 72.130 [69.030,77.600] | 70.625 [67.935,76.100] | 0.481 |
| Gender (%) |  | | |  |  | 0.315 |
|  | Male | | | 38 (53.521) | 12 (66.667) |  |
|  | | Female | | 33 (46.479) | 6 (33.333) |  |
| Education (Median[Q1,Q3]) | | |  | 16.000 [14.000,18.000] | 16.000 [16.000,18.000] | 0.334 |
| BMI (Median[Q1,Q3]) |  | | | 26.251 [23.914,29.296] | 27.353 [23.537,29.530] | 0.683 |
| Hypertension (%) |  | | |  |  | 0.426 |
|  | No | | | 32 (45.070) | 10 (55.556) |  |
|  | Yes | | | 39 (54.930) | 8 (44.444) |  |
| Smoke (%) |  | | |  |  | 0.88 |
|  | No | | | 42 (59.155) | 11 (61.111) |  |
|  | Yes | | | 29 (40.845) | 7 (38.889) |  |
| Drink (%) |  | | |  |  | 0.214 |
|  | No | | | 67 (94.366) | 16 (88.889) |  |
|  | Yes | | | 2 (5.634) | 2 (11.111) |  |
| APOEε4 (%) |  | | |  |  | 0.535 |
|  | No | | | 50 (70.423) | 14 (77.778) |  |
|  | Yes | | | 21 (29.577) | 4 (22.222) |  |
| MMSE (Median[Q1,Q3]) |  | | | 29.000 [27.000,30.000] | 29.000 [28.000,30.000] | 0.566 |

Abbreviation: BMI= Body Mass Index, MMSE= Mini-Mental State Examination score

**Supplementary Table 4：**Final feature sets across all models.

| **Models** | **Features** | |
| --- | --- | --- |
| **Hippocampal radiomics** | **Radiomic- Rad-Score =** 0.381385475* Hippocampus_L_additivegaussiannoise_firstorder_Maximum + 0.354366452* Hippocampus_L_log_firstorder_log-sigma-4-0-mm-3D-Minimum + 0.2991246* Hippocampus_R_wavelet_firstorder_wavelet-LHL-InterquartileRange + 0.2698798* Hippocampus_L_wavelet_glcm_wavelet-LHL-DifferenceVariance + 0.1313076* Hippocampus_L_log_firstorder_log-sigma-1-0-mm-3D-Skewness + 0.100582294* Hippocampus_L_wavelet_ngtdm_wavelet-LHL-Contrast + 0.07689089* Hippocampus_R_additivegaussiannoise_glszm_SizeZoneNonUniformity + 0.063507944* Hippocampus_R_laplaciansharpening_glszm_GrayLevelVariance + 0.05654276* Hippocampus_L_wavelet_glszm_wavelet-LLL-SmallAreaEmphasis + 0.048922736* Hippocampus_R_wavelet_glcm_wavelet-HHH-Idn + 0.032730013* Hippocampus_R_log_glcm_log-sigma-0-5-mm-3D-MaximumProbability + 0.025286676* Hippocampus_L_wavelet_glszm_wavelet-LLH-HighGrayLevelZoneEmphasis + 0.023846291* Hippocampus_L_log_ngtdm_log-sigma-0-5-mm-3D-Complexity + 0.022906903* Hippocampus_R_wavelet_glszm_wavelet-HHH-ZoneVariance + 0.015439521* Hippocampus_L_log_glcm_log-sigma-0-5-mm-3D-ClusterShade + 0.012030174* Hippocampus_L_wavelet_glszm_wavelet-LLL-GrayLevelVariance + 0.003750236* Hippocampus_R_wavelet_glrlm_wavelet-HLL-LowGrayLevelRunEmphasis + 0.003726888* Hippocampus_L_wavelet_gldm_wavelet-LLL-SmallDependenceLowGrayLevelEmphasis + 0.002976302* Hippocampus_R_wavelet_glszm_wavelet-LHL-SmallAreaLowGrayLevelEmphasis + 0.001174081* Hippocampus_L_log_glszm_log-sigma-4-0-mm-3D-GrayLevelNonUniformityNormalized |  |
| **Brain function** | **Function- Rad-Score** = ALFF_Cerebelum_6_R* 0.160701945 + ALFF_Frontal_Mid_Orb_L* 0.1204314 + ALFF_Vermis_3* 0.108492672 + ReHo_ParaHippocampal_L* 0.105556265 + ALFF_Cerebelum_3_R* 0.079803236 + fALFF_Temporal_Pole_Mid_L* 0.066491716 + ALFF_Thalamus_L* 0.0630696 + ALFF_Lingual_R* 0.04335809 | |
| **Brain subregion volume** | volume_Putamen_R, volume_Hippocampus_L, volume_Hippocampus_R, volume_Caudate_L, volume_Caudate_R, volume_Vermis_10, volume_Vermis_9 | |
| **Volume-radiomics** | Radiomic-Rad-Score, volume_Putamen_R, volume_Hippocampus_L, volume_Hippocampus_R, volume_Caudate_L, volume_Caudate_R, volume_Vermis_10, volume_Vermis_9 | |
| **Function- radiomics** | Radiomic-Rad-Score, Function-Rad-Score | |
| **Volume-function** | Function- Rad-Score, volume_Putamen_R, volume_Hippocampus_L, volume_Hippocampus_R, volume_Caudate_L, volume_Caudate_R, volume_Vermis_10, volume_Vermis_9 | |
| **Volume-function-radiomics** | Radiomic- Rad-Score, Function- Rad-Score, volume_Putamen_R, volume_Hippocampus_L, volume_Hippocampus_R, volume_Caudate_L, volume_Caudate_R, volume_Vermis_10, volume_Vermis_9 | |
| **Clinic-volume-radiomics** | Radiomic- Rad-Score, volume_Putamen_R, volume_Hippocampus_L, volume_Hippocampus_R, volume_Caudate_L, volume_Caudate_R, volume_Vermis_10, volume_Vermis_9, age | |
| **Clinic-function-radiomics** | Radiomic- Rad-Score, Function- Rad-Score, age | |
| **Clinic-volume-function** | Function- Rad-Score, volume_Putamen_R, volume_Hippocampus_L, volume_Hippocampus_R, volume_Caudate_L, volume_Caudate_R, volume_Vermis_10, volume_Vermis_9, age | |
| **Clinic-volume-function-radiomics** | Radiomic-Rad-Score, Function-Rad-Score, volume_Putamen_R, volume_Hippocampus_L, volume_Hippocampus_R, volume_Caudate_L, volume_Caudate_R, volume_Vermis_10, volume_Vermis_9, age | |

**Supplementary Figure 1：**The flowchart of patient selection.


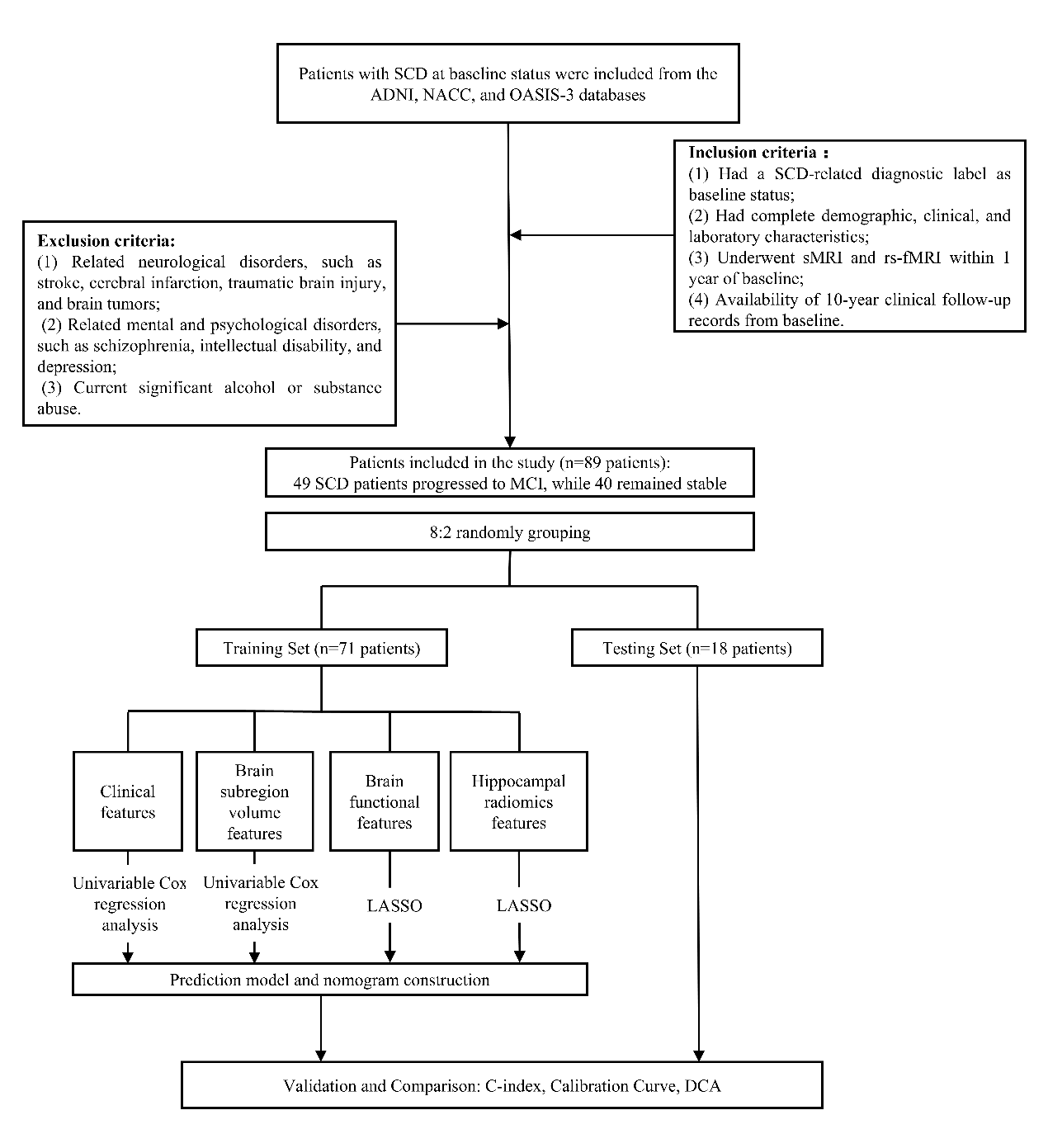


**Supplementary Figure 2：**Kaplan-Meier curve of transition time based on the clinical-volume-function-radiomics model: training set (a) and testing set (b).


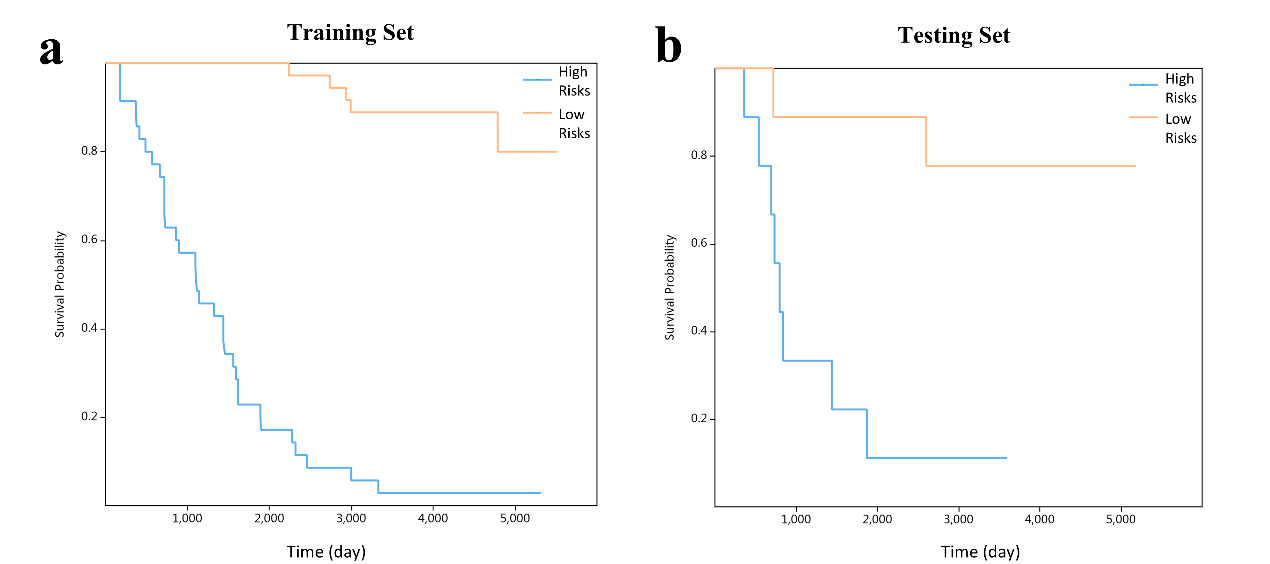

Supplement: Supplementary file 1 [file mmc1.docx]
